# The prognostic and therapeutic potential of HO-1 in leukemia and MDS

**DOI:** 10.1186/s12964-023-01074-8

**Published:** 2023-03-13

**Authors:** Mohammad Sadeghi, Mehrdad Fathi, Jamshid Gholizadeh Navashenaq, Hamed Mohammadi, Mehdi Yousefi, Mohammad Hojjat-Farsangi, Afshin Namdar, Ali Akbar Movasaghpour Akbari, Farhad Jadidi-Niaragh

**Affiliations:** 1 Immunology Research Center, Tabriz University of Medical Sciences, Tabriz, Iran; 2 Student Research Committee, Tabriz University of Medical Sciences, Tabriz, Iran; 3 Noncommunicable Diseases Research Center, Bam University of Medical Sciences, Bam, Iran; 4 Non-Communicable Diseases Research Center, Alborz University of Medical Sciences, Karaj, Iran; 5 Stem Cell Research Center, Tabriz University of Medical Sciences, Tabriz, Iran; 6 Bioclinicum, Department of Oncology-Pathology, Karolinska Institute, Stockholm, Sweden; 7 Department of Immunology, University of Toronto, Toronto, Canada; 8 Hematology and Oncology Research Center, Tabriz University of Medical Sciences, Tabriz, Iran; 9 Department of Immunology, Faculty of Medicine, Tabriz University of Medical Sciences, Tabriz, Iran; 10 Research Center for Integrative Medicine in Aging, Aging Research Institute, Tabriz University of Medical Sciences, Tabriz, Iran

**Keywords:** Heme oxygenase-1, Myelodysplastic syndrome, Leukemia, Hematopoietic malignancies, Chemoresistance

## Abstract

**Background:**

Heme oxygenase-1 (HO-1), a heme-degrading enzyme, is proven to have anti-apoptotic effects in several malignancies. In addition, HO-1 is reported to cause chemoresistance and increase cell survival. Growing evidence indicates that HO-1 contributes to the course of hematological malignancies as well. Here, the expression pattern, prognostic value, and the effect of HO-1 targeting in HMs are discussed.

**Main body:**

According to the recent literature, it was discovered that HO-1 is overexpressed in myelodysplastic syndromes (MDS), chronic myeloid leukemia (CML), acute myeloblastic leukemia (AML), and acute lymphoblastic leukemia (ALL) cells and is associated with high-risk disease. Furthermore, in addition to HO-1 expression by leukemic and MDS cells, CML, AML, and ALL leukemic stem cells express this protein as well, making it a potential target for eliminating minimal residual disease (MRD). Moreover, it was concluded that HO-1 induces tumor progression and prevents apoptosis through various pathways.

**Conclusion:**

HO-1 has great potential in determining the prognosis of leukemia and MDS patients. HO-1 induces resistance to several chemotherapeutic agents as well as tyrosine kinase inhibitors and following its inhibition, chemo-sensitivity increases. Moreover, the exact role of HO-1 in Chronic Lymphocytic Leukemia (CLL) is yet unknown. While findings illustrate that MDS and other leukemic patients could benefit from HO-1 targeting. Future studies can help broaden our knowledge regarding the role of HO-1 in MDS and leukemia.

**Video abstract**

**Supplementary Information:**

The online version contains supplementary material available at 10.1186/s12964-023-01074-8.

## Background

Myelodysplastic Syndromes (MDSs), a group of blood disorders with poorly defined pathophysiology, are known as conditions in which impaired maturation of blood cells in Bone Marrow (BM) is observed, resulting in different degrees of cytopenia in the Peripheral Blood (PB) [1]. Near one-third of MDS progress to Acute Myeloid Leukemia (AML), while the other two-thirds are transformed into high-risk MDS [2].

Leukemia is characterized by the excess of White Blood Cells (WBCs) in the PB and BM of patients [3–5]. Clinically, this disease can be divided into 4 major categories; based on the origin of the affected cells to lymphoid and myeloid and based on the percentage of blasts to chronic and acute leukemia. The major groups of leukemia comprise four common categories including Acute Lymphoblastic Leukemia (ALL), Chronic Lymphoblastic Leukemia (CLL), Chronic Myeloid Leukemia (CML), and AML. Treatment of leukemia depends on the type of disease, the patient's age, and general health. The first-line treatment for all types of leukemia is mainly chemotherapy, except for CML which involves Tyrosine Kinase Inhibitors (TKIs) [6]. However, radiation therapy, Hematopoietic Stem Cell Transplantation (HSCT), targeted therapy, as well as a combination of these approaches may also be utilized. Despite this, due to the high toxicity of the chemotherapeutics [7], and the possibility of rising chemo-resistant clones of malignant cells in many patients, there is a need for novel treatment approaches that efficiently kill chemo-resistant clones and reduce the required dose of the drugs [3, 8, 9]. Numerous factors can contribute to resistance to chemotherapy. A crucial factor is the increased or abnormal expression of Heme Oxygenase-1 (HO-1). HO-1 causes treatment resistance by several mechanisms including the induction of autophagy [10], cell survival, and by its anti-inflammation, and anti-oxidant properties [11], etc. discussed in the following paragraphs [12]. In most solid tumors, this enzyme is known as a main factor leading to treatment resistance [13]. Therefore, numerous studies are also conducted regarding the role of HO-1 in different Hematological Malignancies (HMs).

In this article, the role of HO-1 in MDS and leukemia is reviewed and its prognostic role as well as its targeting effect in different leukemias and MDS is determined.

## Heme oxygenase

Heme oxygenases (HO) are enzymes coded by the *HMOX* genes. HO includes three forms, HO-1 or heat shock protein 32 (Hsp32), which is an inducible form, HO-2, which has a constitutive expression [14], and HO-3 which seems to be a pseudogene derived from HO-2 transcripts [15] and is only expressed in rat neurons [16]. The HO-1 coding gene called *HMOX1* is located on chromosome 22q13.1 and the HO-2 coding gene called *HMOX2* is located on chromosome 16p13.3. Both genes encode enzymes that have the same function [14]. HO-1 expression is induced by a large number of drugs and chemicals including statins, aspirin, niacin, oxidants, inflammatory mediators, heme, [17–19] and physical stimulation (Radiation; ultraviolet for instance [16]). Both enzymes are similar in function, mechanism, cofactors, required substrates, and sensitivity to inhibition by metalloporphyrins, molecules in which the central iron atom is replaced by elements such as tin, zinc, cobalt, and chromium [17–19]. HO catabolizes the heme molecules (the prosthetic group of hemoproteins) into arbon monoxide (CO), biliverdin, and ferrous iron (Fe^2+^) [20]. Heme can produce free radicals by the means of its pro-oxidant catalytic activity of the Fe^2+^ atom, which can act as a cytotoxic agent [21]. Over time, HO-1 activity reduces the number of free heme, therefore, preventing Reactive Oxygen Species (ROS) production, resulting in decreased cell damage. HO-2 (the non-inducible form) is less important than HO-1 in heme catabolism [21, 22].

### HO-1 transcriptional regulation

Oxidative stress is a strong regulator of *HMOX1* transcription through several signaling pathways and transcription factors [23]. Oxidative stress causes the release of heme from the pocket of hemoproteins. Free heme binds to BTB and CNC homolog 1 (Bach1) (a transcription inhibitor of *HMOX1*) and causes a conformational change in its structure, resulting in unbinding of Bach1 from the Stress-Responsive Elements (StREs) of the *HMOX1* gene, leading to increased transcription of HO-1 in the cell. Simultaneously, the increased ROS levels, cause Kelch-like ECH-associated protein 1 (Keap1) detachment from NF-E2-related Factor 2 (Nrf2) allowing it to translocate into the nucleus and bind Antioxidant Response Elements (AREs) of the *HOMX1* gene. Following the release of Bach1 from the StREs of the gene and binding of Nrf2 to the ARE, *HMOX1* transcription is increased [21, 24–27]. Another study reports that HO-1 expression is induced by a ROS/Protein Kinase C (PKC) δ/c-Jun N-terminal Kinase (JNK) 1/2-dependent axis in mouse brain endothelial cells [28]. According to Koyani et al., ROS induction in MG-63 cells promoted P38 Mitogen-Activated Protein Kinases (p38-MAPK) activation and subsequent Protein Kinase B (PKB) phosphorylation which in turn resulted in nuclear expression of Nrf2 and Early growth response protein 1 (Egr1), leading to HO-1 increased transcription [29]. Several other pathways have also been observed to induce HO-1 through Phosphatidylinositol 3-Kinase (PI3K)/AKT, Hypoxia-Inducible Factor-1 alpha (HIF-1α), etc. [30]. Following HIF-1α inhibition, HO-1 induction was suppressed, indicating that HIF-1α is an inducer of HO-1 [31]. Moreover, according to Rushworth et al., HO-1 expression is negatively regulated by NF-КB (P50/NF-КB1 and P65 subunits) in AML cells [32]. Therefore, HO-1 expression is regulated by several factors.

### Roles of HO-1 in protecting cancer cells

Tumor cells have an extremely high need for nutrients to meet their demanding anabolic requirements and energy production [33]. Metabolic plasticity, which can be achieved by changes or mutations in the expression of metabolic genes, is essential for tumor survival [34]. Since malignant cells consume a lot of nutrients, thus, produce large quantities of ROS therefore, HO-1 is involved in the survival of leukemic cells. In addition to cell metabolism, HO-1 appears to play roles in the tumor microenvironment as well. The BM microenvironment, a tissue composed of cellular and non-cellular components, acts as a niche for maintaining and regulating stem cells [35]. The BM microenvironment has been an interesting topic for scientists in recent years since it has been proven that certain changes, even if small, in this tissue can lead to leukemia, and this microenvironment plays role in the pathology of leukemia [34]. In the following, the studies that investigated the role of HO-1 in the leukemic microenvironment have been reviewed. In this section, the role of HO-1 in protecting cancer cells from apoptosis is discussed.

The cytoprotective effects of HO-1 are exerted by itself or mediated by its products [21]. HO-1 activity prevents the apoptosis of cells in response to Tumor Necrosis Factor (TNF) treatment, mediated by detoxifying heme [22]. Besides protection against TNF-mediated apoptosis, HO-1 induces the expression of B-cell lymphoma-extra-large (Bcl-Xl), and B-cell lymphoma-2 (Bcl-2) and inhibits the expression of Bcl-2 associated X (Bax) and Bcl-2 homologous antagonist/killer (Bak) resulting in decreased apoptosis through P38-MAPK and PI3K pathways [36–39]. HO-1 activity is also found to be associated with autophagy, a main mechanism leading to chemoresistance which appears to be mediated by the inhibition of the mammalian Target of Rapamycin (mTOR) activity [10]. Moreover, HO-1 is also reported to induce Enhancer of Zest Homolog 2 (EZH2) expression, an anti-oncogene repressor, resulting in the methylation of anti-oncogenes such as *P53, P15,* and* P16* [40]. Additionally, HO-1 induces Vascular Endothelial Growth Factor (VEGF) and CXCL12 expression by BM stromal cells (BMSCs) through the PI3K/AKT pathway, providing a tumor-nurturing microenvironment [41].

Other tumor-favored functions of HO-1 are exerted by its products [42]. Following the catabolism of heme to Fe^2+^, CO, and biliverdin (which is then converted into bilirubin by a reaction mediated by biliverdin reductase) each of these products exerts different mechanisms to protect the cell. CO, by binding to cytochrome c oxidase induces signaling through the P38-MAPK isoform, resulting in increased cell survival, which resembles the previously mentioned activity of HO-1 [21, 43]. It has also been proven that CO possesses immunomodulatory properties by inhibiting the function of T and dendritic cells [27]. Fe^2+^ atom released from the protoporphyrin ring, compared to the Fe^2+^ atom in the heme pocket, is neutralized by sequestration into the ferritin structure and the activity of the Fe efflux pump, resulting in decreased ROS generation [21, 44–47]. The last product of the enzyme, bilirubin (derived from biliverdin by the activity of biliverdin reductase), is proven to possess strong antioxidant properties [48] at micromolar concentrations [49]. The main anti-oxidant effect of bilirubin results from the biliverdin-bilirubin cycle. A mechanism in which bilirubin is converted to biliverdin by reacting with ROS and therefore neutralizing it, followed by the reduction into bilirubin by the activity of biliverdin reductase [50]. Moreover, bilirubin decreases the expression of P and E selectins [11, 51, 52] therefore, possessing anti-inflammatory properties as well [12, 53]. Thus, HO-1 can increase cell survival from a variety of pathways.

**Fig. 1:**
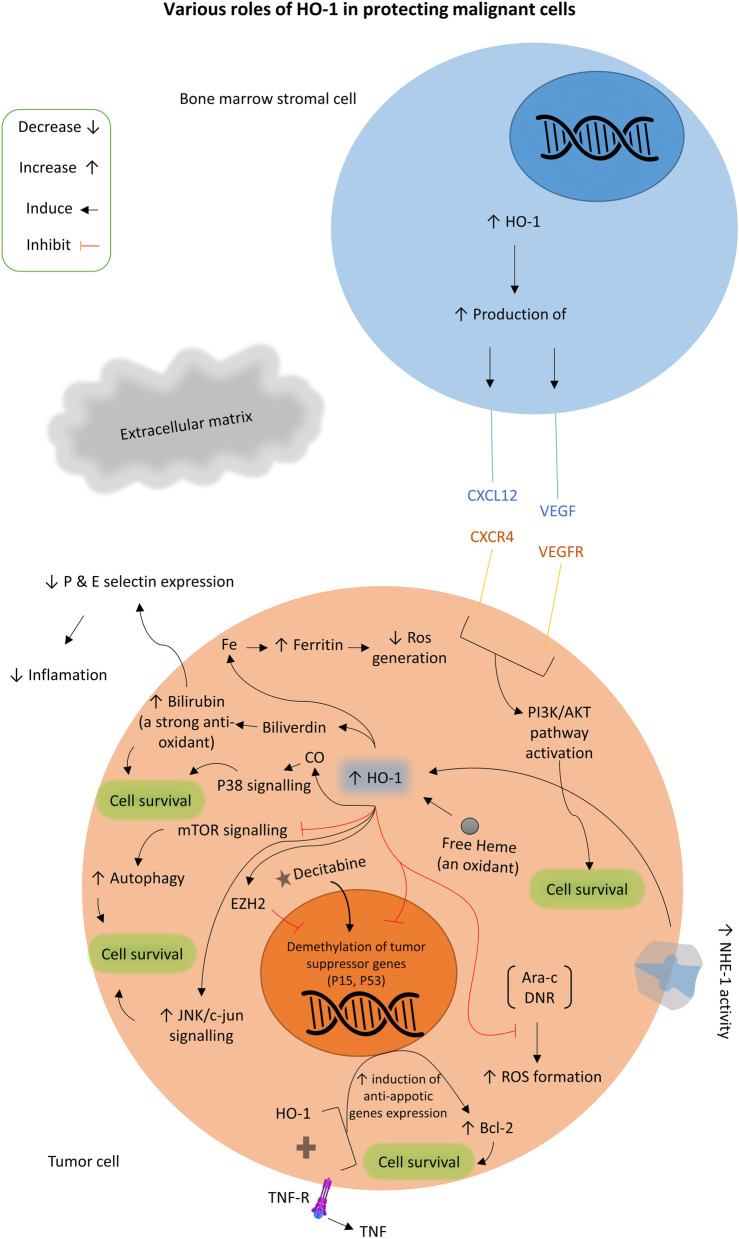
Various roles of HO-1 in protecting malignant cells. An abstract of different roles of HO-1 in malignant as well as in adjacent stromal cells is provided. Ara-c: Cytarabine; Bcl-2: B-cell lymphoma-2; DNR: Daunorubicin; EZH2: Enhancer of Zeste Homolog 2; HO-1: Heme Oxygenase-1; NHE1: Na^+^-H^+^ Exchanger 1; ROS: Reactive Oxygen Species; TNF: Tumor Necrosis Factor; TNFR: TNF Receptor; VEGF: Vascular Endothelial Growth Factor; VEGFR: VEGF Receptor; mTOR: mammalian Target Of Rapamycin

A brief conclusion about the various roles of HO-1 in protecting malignant cells is presented in Fig. 1.

## HO-1 expression pattern and its prognostic value in MDS and leukemia

An elevated level of ROS is generated in cancer cells, which is in part due to the increased metabolism of neoplastic cells. Cancer cells utilize different antioxidant proteins including HO-1 to ensure adaptations to elevated levels of ROS and oxidative stress [54, 55]. Thus, an abnormal or increased expression of HO-1 is predicted to be observed in HMs. Here we review the data regarding the expression of HO-1 in MDS and different leukemias. A brief review of conducted studies on the expression pattern and prognostic value of HO-1 is provided in Table 1.

**Table 1:** HO-1 expression pattern and prognostic value in leukemia and MDS

Disease	Expression status	Expressing cells	Main claim	Ref.
MDS	Increased	BM macrophages	HO-1 overexpression by BM macrophages deteriorates MDS patients' prognoses	[56]
MDS	Increased	SKM-1 cell line and PB cells	HO-1 overexpression is associated with high-risk disease and decitabine resistance	[58]
MDS	Increased	Primary cells	HO-1 overexpression induces EZH2 through pRB-EBF, resulting in p15 and p53 silencing and more progression to AML	[40]
MDS	Increased	SKM-1 cell line and BM cells	HO-1 overexpression is correlated with high-risk MDS	[60]
MDS	Increased	SKM-1 cell line and BM cells	HO-1 overexpression is more frequently seen in high-risk than low-risk MDS and contributes to AZA resistance	[62]
CML	Increased	Primary cells	HO-1 level is strongly correlated with CML progression	[69]
CML	Increased	Primary cells	HO-1 overexpression, induced by BCR::ABL activity, results in IM resistance	[70]
CML	Increased	CML cell line	HO-1 overexpression is correlated with IM resistance in CML	[71]
CML	Increased	Cell lines and primary cells	NHE-1 induces HO-1, through PKC-β phosphorylation and P38-MAPK pathway activation resulting in IM resistance	[73]
CML	Increased	Primary cells	HO-1 induces autophagy, resulting in IM resistance	[10]
CML	Increased	Primary cells	HO-1 overexpression is correlated with IM resistance and HDACs upregulation	[80]
CML	Increased	Primary cells	BCR::ABL induces HO-1 overexpression by a NOX-mediated mechanism, resulting in IM resistance in CML	[81]
CML	increased	BMSCs of CML patients	HO-1 overexpression by BMSCs induces IM resistance	[41]
AML	Increased	Cell line & primary cells	HO-1 overexpression in AML is associated with Ara-c and DNR resistance and increased relapse rate	[30]
AML	Increased	Cell line & primary cells	HO-1 overexpression induces DNR and Ara-c resistance	[82]
AML	Increased	primary cells	HO-1 is overexpressed in AML-M2 cells	[83]
AML	Increased	Cell line & primary cells	HO-1 expression is associated with resistance to Ara-c-induced apoptosis	[84]
AML	Increased	Cell line & primary cells	HO-1 overexpression is correlated with FLT3-ITD expression and poor prognosis	[88]
AML	Increased	Cell line & primary cells	HO-1 is higher in relapsed patients compared to the controls and is correlated with poor prognosis	[90]
AML	Increased	Primary cells	HO-1 overexpression is associated with high-risk disease and decreased OS	[91]
AML	Increased	Primary cells	In addition to leukemic cells, HO-1 is overexpressed in LSCs of AML patients	[92]
AML & ALL	–	Primary cells	HO-1 is a strong predictor of HSCT outcome in AML and ALL	[93]
AML	Increased	Cell line & primary cells	HO-1 overexpression contributes to the immune evasion of AML cells against NK cells	[94]
ALL	Increased	Primary cells	ALL cells, progenitor cells & LSCs of ALL patients strongly express HO-1	[95]
ALL	Increased	Primary cells	HO-1 overexpression in BMSCs of ALL patients induces VEGF expression, therefore, vincristine resistance	[96]
ALL	Increased	Primary cells	IK6 induces HO-1 expression by upregulating STAT5, resulting in ALL poor prognosis and IM-resistance	[99]

*ALL*, acute lymphoblastic leukemia; *AML*, acute myeloid leukemia; *Ara-c*, Cytarabine; *AZA*, 5-Azacytidine; *BM*, bone marrow; *BMSC*, bone marrow stromal cell; *CML*, chronic myeloid leukemia; *DNR*, Daunorubicine; *EZH2*, enhancer of zeste homolog 2; *FLT3-ITD*, Fms-like tyrosine kinase 3-internal tandem duplication; *HDAC*, histone deacetylase; HO-1, heme oxygenase-1; *HSCT*, hematopoietic stem cell transplantation; IK6, dominant-negative Ikaros Isoform 6; *IM*, Imatinib; *LSC*, leukemic stem cell; *MDS*, myelodysplastic syndrome; *OS*, overall survival; *P38-MAPK*, P38 mitogen-activated protein kinases; *PB*, peripheral blood; *PKC-β*, protein kinase C-β; *STAT5*, signal transducer and activator of transcription 5; *TKI*, tyrosine kinase inhibitor; *VEGF*, vascular endothelial growth factor

### MDS

According to several studies, HO-1 expression in MDS is abnormal. In this section, we review the altered expression pattern of HO-1 and its prognostic value in MDS.

Nybakken et al. studied HO-1 expression in MDS patients. MDS patients were categorized into lower-grade and higher-grade MDS. They assessed HO-1 expression in BM macrophages along with other factors such as CD163^+^ macrophage population and iron storage. CD163 is a scavenger receptor for hemoglobin and increases HO-1 expression in CD163^+^ M2 anti-inflammatory macrophages. They found that HO-1 is expressed in CD163-expressing macrophages. Moreover, the CD163^+^ macrophage population along with HO-1 expression was increased in MDS samples compared to benign cytopenic samples. After the follow-up of patients, they found that in MDS, and not in benign cytopenic patients, HO-1 overexpression was associated with lower Overall Survival (OS) [56].

It appears that increased expression of HO-1 by BM macrophages protects MDS cells. Furthermore, it is also possible that iron overload in MDS BM due to the ineffective erythropoiesis and frequent blood transfusions [57], induces HO-1 expression, resulting in increased HO-1 expressing macrophage differentiation, which in turn suppresses erythropoiesis (as these macrophages infiltrate the BM and physically interrupt erythropoiesis) and thus causes more cytopenia. Therefore, HO-1 expression in BM macrophages of MDS patients leads to poor clinical outcomes.

In addition to HO-1 expression by BM macrophages, other studies investigated the expression of HO-1 by MDS cells. Decitabine is an inhibitor of cytosine methylation and is approved for the treatment of high-risk MDS. A study reported the increased expression of HO-1 in MDS cells, which was associated with increased cell viability and resistance to decitabine treatment. Additionally, HO-1 overexpression was correlated with higher-risk disease [58]. Thus, in addition to the prognostic role of HO-1 in MDS, it can be said that HO-1 overexpression is associated with resistance to certain chemotherapeutics such as decitabine.

Enhancer of Zest Homolog 2 (EZH2), the core subunit of Polycomb Repressive Complex 2 (PRC2), is responsible for trimethylating and therefore silencing several target genes. EZH2 is highly dysregulated in cancers [59]. In a study conducted by Wang et al., the role of HO-1 and its association with the EZH2 was studied in MDS. They found higher expression of HO-1 and EZH2 in high-risk MDS patients compared to normal controls. Also, patients who progressed to AML had higher levels of HO-1 compared to other MDS groups. However, no such increased expression was observed in the lower-risk group. They found that HO-1 can stimulate the transcription, as well as the activation of EZH2 through the Retinoblastoma protein (pRB)- Early B-cell Factors (EBF) pathway resulting in the decreased expression of p15^INK4B^ and p53 in MDS cells [40]. The data indicate that HO-1 overexpression in MDS cells is associated with a poor prognosis. Also, it appears that HO-1 overexpression can induce EZH2 activity (through pRB-EBF), which in turn results in decreased expression of anti-tumor genes, which are considered the main targets of decitabine.

Furthermore, Wang et al. in a study on MDS patients showed that both mRNA and protein expression levels of HO-1 were increased compared to controls. HO-1 overexpression was mostly evident in high-risk MDS groups [60].

Another study group investigated the role of HO-1 in resistance to 5-Azacytidine (AZA), a DNA methyltransferase inhibitor used for treating MDS and AML patients [61]. Following the treatment of MDS cells with AZA, HO-1 expression was increased which was correlated with AZA resistance. Also, Bone Marrow Mononuclear Cells (BMMCs) of patients with high-risk disease had significantly higher HO-1 levels compared to the low-risk groups [62]. Thus, it appears that the HO-1 expression pattern is a main factor determining MDS response to AZA and decitabine.

Overall, HO-1 overexpression (either by MDS cells or by BM macrophages) is associated with a higher risk of MDS and more progression to AML. HO-1 through the pRB-EBF pathway blocks the demethylation of p15^INK4B^, which is an important tumor suppressor. Clinical trials targeting HO-1 in combination with decitabine or AZA are needed to prove this in practice. Also, due to the integrity of the data from the mentioned studies, it is safe to state that HO-1 possesses high prognostic value in MDS and can help determine the response to conventional treatments such as AZA, and decitabine.

### CML

CML, a stem cell disease, is known to be associated with the presence of the Philadelphia chromosome (Ph), a result of chromosomal translocation t(9;22) [63, 64]. In the mentioned translocation, the *c-ABL* gene on chromosome 9 is fused to the *BCR* gene on chromosome 22, resulting in *BCR::ABL1* oncogene formation, which encodes for the BCR-ABL1 protein (a constantly active tyrosine kinase) [65–67]. The constant activity of BCR-ABL1 tyrosine kinase induces proliferation and decreases apoptosis in leukemic cells [64]. The main therapy for CML, Imatinib (IM), a Tyrosine Kinase Inhibitor (TKI), is associated with resistance occurring in approximately 40% of patients [6, 68]. Several mechanisms are proposed for this resistance. Many researchers have reported that HO-1 is involved in IM resistance.

Wei et al. investigated the correlation between HO-1 expression and CML progression. They found that the HO-1 mRNA level was significantly higher in patients compared to the healthy controls. Moreover, the HO-1 level rose when relapse occurred. Finally, the expression of HO-1 was strongly correlated with the disease progression, indicating that HO-1 possesses a valuable prognostic value in CML [69].

According to a study performed by Mayerhofer et al. on the role of HO-1 in CML, increased mRNA and protein levels of HO-1 were reported in patients. They found that HO-1 transcription was induced by BCR-ABL tyrosine kinase activity. HO-1 constitutive expression by CML cells increased cell survival in response to IM. Further, HO-1 overexpression seemed to counteract the apoptosis induced by IM [70].

Tibullo et al., also showed that HO-1 mRNA level was significantly higher in IM-resistant cells compared to IM-sensitive cells [71].

According to these studies, HO-1 overexpression in CML cells causes IM resistance by several pathways that are discussed in the following. A possible explanation for the induction of HO-1 expression by BCR-ABL tyrosine kinase could be due to the increased production of ROS in *BCR*: *ABL*^+^ cells, resulting from the increased metabolism of these cells [72]. The elevation in ROS levels induces HO-1 expression. For the same reason, BCR-ABL blocking by IM reduces HO-1 expression in CML cells.

In another study, Ma et al. evaluated the contribution of Na^+^–H^+^ exchanger 1 (NHE1) to HO-1-mediated IM resistance in CML cells. They found excess mRNA and protein expression levels of HO-1 in IM-resistant cells compared to IM-sensitive cells, which was correlated with a higher PH_i_ index. They found that NHE1 activation resulted in Protein Kinase C- β (PKC- β) phosphorylation and P38-MAPK pathway activation, which in turn upregulated HO-1 [73]. The study was continued by targeting these genes, discussed in the following sections.

Autophagy is a natural way of removing unnecessary and dysfunctional proteins and organelles through lysosome-dependent mechanisms [74, 75]. It has been demonstrated that autophagy has an important role in inducing chemoresistance [76]. A protein named mTOR is crucial for autophagy regulation. Accordingly, Cao et al. evaluated the correlation between HO-1 and autophagy in CML resistance to IM. They showed that the mRNA and the protein expression of HO-1 were higher in IM-resistant CML cells compared to non-resistant cells. In addition, IM-resistant cells had higher levels of HO-1 and lower levels of mTOR expression, which was accompanied by Light Chain-3 (LC3)-I/-II autophagy-related proteins expression and elevated levels of autophagy occurring in cells, indicating that HO-1 induces autophagy, resulting in IM resistance [10]. Therefore, another mechanism by which HO-1 desensitizes CML cells to IM is the induction of autophagy.

Histone deacetylases (HDACs) are enzymes that remove acetyl groups from N-acetyl lysine of histones and non-histone proteins [77, 78]. HDAC overexpression in malignant cells is associated with increased tumor survival, proliferation, and angiogenesis [79]. In a study by Wei et al., on the role of HO-1 in IM resistance, higher mRNA and protein levels of HO-1 and HDAC in IM-resistant cells compared to IM-sensitive cells were found. Also, HO-1 expression in CML cells correlated with obvious HDACs upregulation [80]. Therefore, HO-1 is co-expressed with other informants of poor prognosis such as HDACs.

In another study, the role of nicotinamide adenine dinucleotide phosphate (NADPH) oxidase (NOX) was evaluated in HO-1-mediated resistance to IM. Higher levels of HO-1 expression were observed in CML patients with blast crisis compared to patients with chronic or accelerated phase CML. Thus, HO-1 overexpression is associated with a higher risk of disease and poor prognosis. Moreover, *BCR*: *ABL*^+^ cells expressed higher levels of HO-1 compared to *BCR*: *ABL*^*−*^ cells. They found that BCR-ABL, by upregulating the 47 kD cytosolic subunit of NOX (p47phox), increases ROS generation, which in turn leads to increased HO-1 expression and thus IM resistance [81]. Therefore, it can be confirmed that BCR: ABL is a strong inducer of HO-1 expression and one can expect that targeting HO-1 in *BCR::ABL*^+^ CML cells may be more beneficial than in *BCR*: *ABL*^*−*^ CML cells.

In addition to leukemic cells, BMSCs of CML patients also show an increased HO-1 expression. Liu et al. investigated the role of HO-1 expression by BMSCs in IM resistance. The mRNA and protein expression levels of HO-1 in BMSCs of IM-resistant patients were higher than in IM-sensitive patients [41]. They also targeted HO-1 in BMSCs and proposed a mechanism, which we discussed in the targeting section.

Considering the consistency of the obtained data, it is concluded that HO-1 in CML cells is overexpressed by both CML cells and BMSCs of CML patients compared to healthy controls and that BCR-ABL activity induces HO-1 expression either by regulating NOX2 complex or induction of ROS formation due to increased metabolism. HO-1 increased levels lead to IM resistance through various pathways such as induction of HDACs, autophagy induction, and self-mediated functions of the HO-1, thereby, weakening the patient's prognosis.

### AML

Several studies have investigated the expression pattern of HO-1 in AML, which are discussed in this section.

Zhe et al. investigated the role of HO-1 in AML cells. They found higher levels of HO-1 expression both at mRNA and protein levels in Cytarabine (Ara-c) (the main chemotherapy regimen for AML along with Daunorubicin (DNR))-resistant cells compared to Ara-c-sensitive cells. Moreover, high HO-1-expressing cells were resistant to apoptosis induced by Ara-c and DNR. HO-1 expression levels in relapsed patients were higher compared to patients with complete remission and controls. Patients' cells with high HO-1 expression also co-expressed high levels of HIF-1 and glucose transporter-1 (GLUT1) [30].

A study reported that treating AML cells with Ara-c and DNR, increased HO-1 mRNA and protein expression. Also, it was discovered that Ara-c and DNR-mediated apoptosis induction was dependent up to certain levels on ROS formation, and HO-1 induction limited the response to these chemotherapeutics [82]. Similarly, patients with AML (M2) had higher levels of HO-1 expression compared to controls [83].

Also, another study reported that AML patients had higher levels of HO-1 mRNA and protein expression compared to controls. AML-M5 patients were reported to have elevated levels of HO-1 compared to other subtypes. HO-1 overexpression was correlated with leukocytosis. Following cell incubation with Ara-c, cells with high HO-1 expression had lower apoptosis rates and lower caspase 3, 8, and 9 expressions. Also, HO-1 expression increased after Ara-c treatment [84].

According to the studies, it can be confirmed that HO-1 overexpression contributes to Ara-c and DNR resistance. Moreover, HO-1 expression is induced in response to Ara-c and DNR treatment (the main chemotherapy regimens of AML patients), probably through a ROS-dependent pathway. This could be utilized as a means by AML cells to escape chemotherapy-induced apoptosis. Moreover, it may be possible that HO-1 expression is heterogeneous by different AML subtypes, leading to heterogeneous responses to HO-1 targeting.

The most common mutation in AML, Fms-Like Tyrosine kinase receptor 3 (*FLT3*) gene Internal Tandem Duplication (ITD) is associated with poor prognosis [85, 86]. It has been proven that the constant activity of FLT3-ITD produces elevated levels of ROS in the cells, which in turn induces the expression of HO-1 [87]. Kannan et al. evaluated the role of HO-1 in *FLT3-ITD*^+^ AML cells. They found that HO-1 expression was higher in *FLT-ITD*^+^ cells compared to FLT3-wild type AML cells. Moreover, patients with higher levels of HO-1 in AML cells had decreased survival compared to the patients with lower HO-1 levels. The overexpression of HO-1 was associated with quizartinib (FLT3 inhibitor) resistance [88].

Growth Factor Independent-1 (GFI-1) (a transcriptional repressor) inhibits the acetylation of target genes and is proven to prevent several malignancies. Low GFI-1 expression is associated with myeloid malignancies and resistance to HDAC inhibitors [89]. The correlation between GFI-1 and HO-1 expression in AML patients was evaluated by Cheng et al. They found that HO-1 expression in relapsed patients' samples was higher compared to patients with complete remission or healthy controls, and was associated with HDAC1, HDAC2, and HDAC3 upregulation and was reversely correlated with GFI-1 expression. HO-1 overexpression was strongly associated with inferior prognosis and Panobinostat (a pan-HDAC inhibitor) resistance. It was discovered that GFI-1 low expression upregulates HO-1 through a PI3K/AKT pathway resulting in Panobinostat resistance [90].

Also, the prognostic value of HO-1 expression was investigated in another study. The study reported that HO-1 expression was directly correlated with patients' prognosis, as high-risk patients expressed higher levels of HO-1 mRNA and protein compared to other groups. After 3 years of patient follow-up, HO-1 overexpression was associated with decreased OS and relapse-free survival [91].

According to the mentioned studies, it can be concluded that HO-1 is overexpressed in AML cells and is strongly correlated with poor prognosis and high-risk disease in AML.

Other researchers studied the expression of HO-1 in stem cells and BM cells of AML patients. Accordingly, CD34^+^CD38^+^ and CD34^+^CD38^−^ cells of AML patients were reported to express higher levels of HO-1 mRNA and protein compared to normal CD34^+^ cells [92]. The findings of the research suggest that HO-1 can be used as a novel target to help eliminate Minimal Residual Disease (MRD) in AML patients since it is overexpressed in Leukemic Stem Cells (LSCs).

Lue et al. also evaluated the role of HO-1 in HSCT prognosis in BMMCs of AML and ALL patients. The research reported that HO-1 expression was higher in BMMCs of patients with relapse compared to non-relapsed patients. Moreover, they found that patients with Acute Graft Versus Host Disease (aGVHD) had lower levels of HO-1 expression in BMMCs than those without aGVHD pre-HSCT. However, increased HO-1 expression post-HSCT was associated with aGVHD [93].

This study indicates that HO-1 could be used as a strong predictor of relapse and aGVHD following HSCT. Moreover, it is confirmed that LSCs of AML patients overexpress HO-1 as well, which contributes in determining the outcome of HSCT in acute leukemia.

In addition to the effect of HO-1 expression on chemoresistance, a study indicates that HO-1 contributes to the immune evasion of AML cells as well. According to Zhang et al., HO-1 expression level is higher in Relapsed compared to newly diagnosed AML patients' cells and healthy controls. HO-1 overexpression in AML cells was associated with suppressed CD48 (a ligand for 2B4 receptor on Natural Killer (NK) cells) expression levels. When co-culturing with NK cells, AML cells with upregulated HO-1 had higher survival compared to cells with low HO-1 levels. The results indicated that HO-1 suppresses CD48 expression, therefore, suppressing NK cell anti-tumor activity [94].

In conclusion, HO-1 has increased expression in AML cells and causes resistance to several chemotherapeutics including Ara-c, DNR, quizartinib, and HDAC inhibitors (panobinostat in particular). The high expression level of this protein is associated with a poor prognosis in AML patients. Also, concluding from the two previous studies, it can be said that not only does HO-1 expression by the leukemic stem and progenitor cells of AML patients makes this gene a good target for treating MRD, but also has prognostic value in determining the response to HSCT. Moreover, it has been discovered that HO-1 contributes to immune evasion by AML cells, further deteriorating the prognosis of AML patients.

### ALL

In this section, the conducted studies regarding the association between the HO-1 expression pattern and ALL prognoses are provided.

Reiterer et al. reported that HO-1 mRNA and protein expression was present both in (Ph)^+^ and (Ph)^–^ primary cells of ALL patients, as well as CD34^+^CD38^−^ and CD34^+^CD38^+^ stem cells and progenitors. In Ph^+^ cells unlike Ph^−^ cells, IM treatment decreased HO-1 mRNA expression [95]. Therefore, due to the expression of HO-1 by the LSCs and progenitor cells of AML patients, HO-1 targeting could be beneficial in treating MRD.

In addition to leukemic cells, BMSCs of ALL patients express HO-1. Yu et al. also analyzed the HO-1 expression pattern in BMSCs of ALL patients. They found that HO-1 expression was higher in ALL patients compared to normal controls. Also, vincristine (a plant alkaloid) -resistant patients had higher HO-1 expression in BMSCs compared to non-resistant patients. The study suggested that HO-1 overexpression in BMSCs induced VEGF expression, mediated by the PI3K/AKT pathway, resulting in vincristine resistance [96].

Dominant-negative Ikaros Isoform 6 (IK6), a short transcript of the *IKZF1* gene, lacking coding exons 3–6, is a transcriptional repressor [97], reported to disrupts the differentiation and proliferation of lymphoid cells, therefore, possessing leukemogenic potential [98]. A study evaluated the correlation between HO-1 and IK6 and *BCR::ABL*^+^ in ALL patients and showed that most of *BCR::ABL*^+^ ALL patients are IK6^+^. The presence of IK6 in ALL cells was associated with higher WBC count, higher MRD, and a poor clinical response indicating an association between IK6 expression and prognosis. Also, IK6^+^ ALL cells expressed HO-1 four-fold more than IK6^−^ cells. Moreover, a strong correlation between the expression of IK6 and HO-1 was detected, which was associated with IM resistance. It was discovered that IK6 upregulates the Signal Transducer and Activator of transcription 5 (STAT5) which induces HO-1 upregulation, resulting in IM resistance [99]. Therefore, the coexpression of IK6 and HO-1 is associated with IM resistance in ALL. Moreover, HO-1 is coexpressed with other indicators of poor prognosis in ALL.

A study suggests that certain *HMOX1* gene polymorphisms are involved in ALL disease course. This group suggests that the presence of (GT)n repeat sequences in the promoter of *HMOX1* is more frequently found in ALL patients compared to controls and is associated with lower response to treatment, and increased probability of post-chemotherapy neutropenia occurrence. It seems that HO-1 gene polymorphisms could be used as prognostic markers and should further be studied [100].

Altogether, HO-1 overexpression is present in leukemic cells, LSCs, and BMSCs of ALL patients and is associated with a poor prognosis. Moreover, HO-1 is a strong contributor to IM and vincristine resistance. Also, it appears that the presence of certain HO-1 polymorphisms could be used as important prognostic determinants.

## Targeting HO-1 in MDS and leukemia

Regarding the therapeutic value of HO-1 in MDS and leukemia, several studies have tried to target it in HMs. Here, we tried to summarize the most important findings in this field. A brief conclusion of the studies is presented in Table 2.

**Table 2:** Targeting HO-1 in MDS and leukemia

Disease	Type of study	Samples	Method of HO-1 inhibition	Main claim	Ref.
MDS	Ex vivo	SKM-1 cell line & 41 patient sample	siRNA	HO-1 silencing synergizes with decitabine in demethylating anti-oncogenes	[58]
MDS	Ex vivo	58 patient sample	siRNA	HO-1 silencing induces p15^INK4B^ expression and reverses decitabine resistance	[40]
MDS	Ex vivo	SKM-1 cell line	siRNA	HO-1 silencing reduces DNMT1, overcoming decitabine resistance	[102]
MDS	Ex vivo*/ *in vivo	SKM-1 cell line & 48 patient sample	siRNA	HO-1 silencing reduces DNMT1 levels resulting in cell cycle arrest in MDS cells and increased sensitivity to AZA-induced apoptosis	[62]
MDS	Ex vivo	SKM-1 cell line & 32 patient sample	ZnPP	HO-1 blocking exerts direct apoptotic effects sensitizing MDS cells to 4SC-202	[60]
CML	Ex vivo	K562 cell line & 15 patient sample	ZnPP	HO-1 blocking induces apoptosis mediated by its products-independent mechanism	[70]
CML	Ex vivo	K562 & LAMA 84 cell line	siRNA	The translocation of HO-1 into the nucleus induces IM resistance	[71]
CML	Ex vivo	K562/K562R cell line & 12 patient sample	SiRNA/ZnPP	HO-1 inhibits mTOR, inducing autophagy and IM resistance	[10]
CML	Ex vivo*/ *in vivo	K562 cell line & 23 patient sample	SiRNA/ZnPP	HO-1 inhibition synergies with IM in eliminating CML cells	[103]
CML	Ex vivo	K562 cell line & 30 patient sample	siRNA	HO-1 silencing in BMSCs sensitizes CML cells to IM by inhibiting VEGF and CXCL12 expression by BMSCs	[41]
CML	Ex vivo	K562/K562R cell line & 35 patient sample	ZnPP	Co-inhibition of NHE1 and HO-1 could further sensitize cells to IM	[73]
CML	Ex vivo	K562/K562R cell line & 35 patient sample	siRNA	HO-1 silencing results in HDACs downregulation and increased sensitivity to IM	[80]
CML	Ex vivo	K562R cell line	HO-1/IMI hybrids	IM combined with HO-1 inhibition overcomes IM resistance	[104]
CML	Ex vivo	K562R cell line	HO-1/NILI hybrids	The study suggests that HO-1 is not involved in NIL resistance	[105]
AML	Ex vivo	U937 & HL-60R cell line & patient sample	siRNA	Silencing HO-1 overcomes Ara-c resistance	[30]
AML	Ex vivo	U937, HL60, & K562 cell line & 11 patient sample	miRNA	HO-1 protects AML cells from ROS accumulation induced by Ara-c and DNR	[82]
AML	Ex vivo*/*in vivo	Kasumi-1 cell line & 15 patient sample	siRNA	HO-1 silencing induces caspase-3, 8, 9, dependent apoptosis in response to DNR	[83]
AML	Ex vivo*/*in vivo	U937 & THP-1 & Kasumi-1 cell line & patient sample	siRNA	HO-1 activates the JNK/c-jun pathway to protect AML cells against Ara-c	[84]
AML	Ex vivo*/*in vivo	HL60, U937 & KG1 cell line & 58 patient sample	ZnPP	HO-1 blockade in AML cells and LSCs synergizes with Ara-c in inducing apoptosis	[92]
AML	Ex vivo	U937, HL60, & K562 cell line & 16 AL patient sample	siRNA	HO-1 silencing sensitized AML cells to Ara-c	[107]
AML	Ex vivo	Kasumi-1 cell line	siRNA	HO-1 silencing sensitizes AML cells to DNR by inducing both mitochondrial and endoplasmic-dependent apoptosis inductions	[108]
AML	Ex vivo	HL-60, THP-1, NB4, & NB4-R2 cell lines	ZnPP	HO-1 induces resistance to ATO, while ATRA treatment overcomes it by reducing Nrf2 expression	[109]
AML	Ex vivo*/*in vivo	KG-1, THP-1, MOLM13, MOLM13-TKIR, MV411 cell lines & 18 primary samples	siRNA/ZnPP	HO-1 silencing synergizes with quizartinib in eliminating FLT3-ITD^+^ quizartinib-resistant/-sensitive cells	[88]
AML	Ex vivo*/*	Kasumi-1 and HL-60 cell lines and 64 patient samples	siRNA	HO-1 silencing overcomes HDAC inhibitor resistance in AML (even in low GFI-1 expressing cells)	[90]
AML	Ex vivo*/*in vivo	Kasumi-1 cell line	SiRNA/ZnPP	UA in combination with HO-1 silencing induces the same apoptotic response as Ara-c	[110]
AML	Ex vivo*/*in vivo	THP-1, U937, MV4-11, K562, HL60, and HEK293T and 40 BM patient samples	siRNA	HO-1 upregulation induces Sirt1, which in turn inhibits CD48 expression resulting in NK-cell anti-tumor activity inhibition	[94]
AML	Ex vivo	THP-1 cell line	siRNA/ ZnPP/ CuPP	NF- КB co-inhibition with HO-1 sensitizes apoptosis-resistant cells to TNF	[32]
AML	Ex vivo	THP-1, HL60, U937, & AML 193 cell line	siRNA	NF-КB should be co-inhibited with HO-1 to prevent apoptosis-resistance of AML cells	[115]
AML	Ex vivo	THP-1 & HL-60 cell line & patient sample	siRNA	Combinational inhibition of HO-1 and FLIP_L_ induces further apoptotic signals in AML cells in response to TNF compared to the mono-silencing of each gene	[116]
CLL	Ex vivo	MEC1 cell line	SiRNA/ ZnPP	HO-1 blocking induces MMP-9 which in turn results in ATO and probably Fludarabine resistance	[119]
ATL	Ex vivo	TaY cell line	ZnPP	HO-1 inhibition induces bortezomib resistance in ATL cells	[120]
CLL	Ex vivo	MEC1 cell line	shRNA/ ZnBG	HO-1 silencing sensitizes CLL cells to auranofin by a ROS-dependent mechanism	[121]
ALL	Ex vivo	Several cell lines & 26 patient sample	ZnPP/ siRNA	HO-1 silencing sensitizes ALL cells to bendamustine and IM	[95]
ALL	Ex vivo	SupB15 cell line & 42 patient BM CD34 + cells	siRNA	Targeting HO-1 can attenuate the negative effect of IK6 in Ph + ALL patients	[99]
ALL	Ex vivo*/*in vivo	CCRF-SB & Sup-B15 cell line & patient sample	siRNA	HO-1 silencing in BMSCs sensitizes co-cultured ALL cells to vincristine	[96]
ALL	Ex vivo	34 newly diagnosed ALL sample	ZnPP	Silencing HO-1 sensitizes ALL cells to LMK-235 by a Smad7-dependent pathway	[123]

*ALL*, acute lymphoblastic leukemia; *AML*, acute myeloid leukemia; *Ara-c*, Cytarabine; *ATL*, adult T-cell leukemia; *ATO*, arsenic trioxide; *ATRA*, all-trans retinoic acid; *AZA*, 5-azacytidine; *BMSC*, bone marrow stromal cell; *CLL*, chronic lymphocytic leukemia; *CML*, chronic myeloid leukemia; *CuPP*, cupper-protoporphyrin; *CXCL12*, C-X-C motif chemokine 12; *DNMT1*, DNA methyltransferase 1; *DNR*, Daunorubicine; *FLIP*_*L*_, FLIC-like inhibiting protein long form; *FLT3-ITD*, Fms-like tyrosine kinase 3-internal tandem duplication; *GFI-1*, growth factor independent-1; *HDAC*, histone deacetylase; *HO-1*, heme oxygenase-1; *IK6*, ikaros isoform 6; *IM*, Imatinib; *IMI*, imatinib inhibitor; *JNK*, Jun N-terminal kinase; *MDS*, myelodysplastic syndrome; *MMP-9*, matrix metalloproteinase-9; *mTOR*, mammalian target of rapamycin; *NF-КB*, nuclear factor kappa-light-chain-enhancer of activated B cells; *NHE1*, Na^+^–H^+^ exchanger 1; *NIL*, nilotinib; *NILI*, nilotinib inhibitor; *Nrf-2*, nuclear factor erythroid 2–related factor-2; *Ph*, philadelphia chromosome; *ROS*, reactive oxygen species; *shRNA*, short hairpin RNA; *SiRNA*, small interfering RNA; *Sirt1*, silent information regulator 1; *Smad7*, suppressor of mothers against decapentaplegic 7; *TKI*, tyrosine kinase inhibitor; *TNF*, tumor necrosis factor; *UA*, ursolic acid; *VEGF*, vascular endothelial growth factor; *ZnBG*, zinc deuterophyrin IX 2, 4-Bis-ethylene glycol; *ZNPP*, zinc protoporphyrin

### Targeting HO-1 in MDS

Due to the increased expression of HO-1 in MDS and its association with patient prognosis, targeting it in MDS has been an interesting topic for researchers. As mentioned previously, decitabine treatment aims to demethylate anti-tumor genes to enhance apoptosis. *P15*^*INK4B*^, an anti-tumor gene, when hyper-methylated is an indicator of MDS transformation to AML and a more blastic BM [101]. In the continuum of their study Ma et al., upregulated HO-1 expression in MDS cells, which was associated with increased proliferation and resistance to apoptosis in response to decitabine. On the other hand, HO-1 down-regulation led to significant demethylation of the *p15*^*INK4B*^ gene, induced by decitabine and induction of apoptosis. While, the blocking caspase-3 pathway inhibited apoptosis by p15^INK4B^, indicating that HO-1 blocking induces p15^INK4B^ and other pro-apoptotic proteins resulting in caspase 3 activation, thus programmed cell death [58].

Wang et al. also demonstrated that the expression of HO-1 and EZH2 is increased in MDS cells and HO-1 could stimulate the transcription and activation of EZH2 through a pRB-EBF-dependent pathway in MDS cells. EZH2 and HO-1 increased expression was associated with the decreased levels of p15^INK4B^ and p53 in MDS cells. Following HO-1 silencing, p15^INK4B^, and p53 expression were increased in response to decitabine. Moreover, they suggested that MDS cells did not respond well to decitabine treatment because of the increased levels of HO-1 which induced EZH2 and in turn reduced p53 and p15^INK4B^ expression in MDS cells [40].

Another study also evaluated the role of HO-1 in decitabine resistance. Following HO-1 upregulation, cell sensitivity to decitabine decreased. Also, HO-1 increased expression was accompanied by p15 decreased levels and demethylation by decitabine, which was reversed by HO-1 silencing. Additionally, they reported that DNA methyltransferase-1 (DNMT1) activity was involved in the decreased demethylation effect of decitabine. They suggested that HO-1 through DNMT1 reduces p15 expression, as DNMT1 blocking attenuated the effect of HO-1 on p15 expression and HO-1 inhibition reduced the expression of DNMT1 in MDS cells. Additionally, HO-1 silencing combined with DNMT-1 inhibition induced further apoptosis in response to decitabine [102].

Thus, HO-1 through EZH2, DNMT1, and possibly other proteins, reduces the expression of p15^INK4B^ and P53, resulting in decreased apoptosis in response to decitabine treatment. It can be concluded that HO-1 inhibition, in combination with decitabine, can overcome decitabine resistance and enhance the treatment result.

In another investigation, HO-1 inhibition significantly inhibited MDS cell growth and increased the apoptosis rate in AZA-treated cells compared to controls. Following HO-1 inhibition, the expression levels of caspase 3 and 9 increased, while the expression of BcL-2 was decreased. HO-1 induction by hemin induced MDS cell cycle progression to the G2/M phase, while its inhibition was accompanied by G0/G1 arrest. HO-1 induction by hemin decreased the expression of p16, and p15 and increased the expression of BcL-2 and DNMT1. In vivo models of MDS established from MDS cell-injected mice demonstrated that HO-1 silenced cell recipient mice had increased OS compared to those without HO-1 silencing [62].

According to this study, it is evident that HO-1 induces DNMT1 expression resulting in AZA-chemoresistance. Further studies simultaneously targeting HO-1 and DNMT1 in combination with AZA appear to be helpful.

Wang et al. also studied the role of HO-1 in resistance to 4Sc-202. 4Sc-202, an inhibitor of histone lysine-specific demethylase 1 and class 1 histone deacetylase, is considered to be a potential therapeutic agent for the treatment of MDS. They found that HO-1 has the potential to reverse the effect of 4Sc-202 in inducing apoptosis in MDS cells. Following HO-1 upregulation by lentivirus, 4Sc-202 failed to induce apoptosis in the cells. Cells with HO-1 overexpression were injected into the mice and results were compared to mice injected with control MDS cells. Decreased apoptosis and survival in HO-1 overexpressed MDS cell recipients in response to 4Sc-202 treatment were observed. They found that HO-1 significantly reversed BcL-2 down-regulation by 4Sc-202. Also, HO-1 decreased the upregulation of cleaved caspases 3 and 9. Following the inhibition of HO-1, the rate of apoptosis increased in MDS cells [60].

Overall, according to data we obtained from several studies, HO-1 mainly functions as an inhibitor of DNA demethylation in MDS. In addition, in vivo studies support the fact that HO-1 is an inducer of BcL-2 expression. HO-1 blockade induces the demethylations of anti-oncogenes (including p15, p16, and p53), resulting in improved elimination of MDS cells in response to decitabine and AZA.

### Targeting HO-1 in CML

Due to the proven role of HO-1 in inducing resistance to IM, its targeting effect in CML has been studied by many researchers.

According to a study, BCR-ABL oncoprotein upregulates HO-1 transcription. Moreover, HO-1 induction by hemin decreased apoptosis rates in response to IM. Following HO-1 inhibition, CML cell viability decreased. They also found that HO-1 mediates its antiapoptotic functions apart from its enzymatic products, as single or combinational treatment of cells with CO, biliverdin, and Fe did not significantly enhance the survival of CML cells in response to IM treatment [70]. Thus, other mechanisms by HO-1 seem to intervene in apoptosis induction.

In another investigation, Tibullo et al. evaluated the role of HO-1 in resistance to IM in CML. Treating cells with IM did not significantly affect the expression of HO-1 mRNA, which contradicts the data of the previous study. However, treating cells with hemin resulted in IM-induced-apoptosis failure. Following HO-1 silencing, IM resistance was reversed. Treatment of CML cells with hemin resulted in significantly decreased ROS production. It was discovered that HO-1 by translocation into the nucleus of the cell and inhibition of ROS accumulation, exerts its anti-apoptotic effects, and its products are not involved in resistance induction to IM, as inhibition of HO-1 migration into the nucleus resulted in decreased cell protection [71].

Based on the two previous studies, it is concluded that HO-1 possesses other unknown anti-apoptotic functions apart from its known enzymatic activity. For instance, HO-1 migration to the nucleus of cells induces TKI resistance mediated by an unknown mechanism.

In one study, following HO-1 upregulation, autophagy and autophagy-related proteins increased; however, HO-1 silencing reversed this effect, which was associated with the activation of the mTOR pathway. Following HO-1 blocking, autophagy-related proteins such as LC3I/II decreased, while mTOR protein expression was increased and inhibited autophagy. HO-1 exerts the same effect on autophagy as rapamycin (an mTOR inhibitor). Moreover, it was discovered that an increase in HO-1 and autophagy occurrence was associated with IM resistance [10]. Thus, HO-1 induces autophagy by inhibiting the mTOR pathway, leading to IM resistance.

In another attempt for targeting HO-1, Mayerhofer et al. silenced HO-1 in CML cells, which led to decreased cell viability. Moreover, the treatment of cells with Zinc Protoporphyrin (ZnPP) (an HO-1 inhibitor), coupled either to polyethylene glycol (PEG-ZnPP) or to a copolymer of styrene-maleic acid (SMA-ZnPP), decreased cell proliferation. Additionally, CD34^+^/ progenitor cells of CML patients in response to ZnPP treatment also showed decreased growth. Moreover, IM-resistant CML cells in response to ZnPP treatment demonstrated decreased proliferation. Treatment of mice injected with IM-resistant CML cells significantly decreased tumor growth. They found that treating CML cells with ZnPP induces apoptosis. They also investigated the effect of HO-1 inhibition combined with IM treatment and found a strong synergism between the treatments. Also, the rate of apoptosis was significantly higher in the combinational group when compared to the groups subjected to monotherapies [103]. According to this study, HO-1 blockade decreases the growth of CML and CML LSCs in response to IM.

As stated previously, HO-1 overexpression in BMSCs of CML patients induces IM resistance [41]. Liu et al. aimed to investigate the mechanism behind this process. Following HO-1 upregulating in BMSCs of CML patients, cocultured with CML cells, increased survival of CML cells due to more VEGF and CXCL12 expression by BMSCs was observed. They found that the binding of CXCL12 and VEGF to their receptor resulted in the activation of the PI3K/AKT pathway in leukemic cells leading to BcL-2 induction and therefore decreased apoptosis in response to IM. Interestingly, following HO-1 downregulation in BMSC, BcL-2 expression decreased in CML cells [41]. According to these studies, HO-1 is overexpressed by both LSCs and BMSCs (BM tumor microenvironment). Therefore, making HO-1 an ideal target for eliminating MRD in CML patients.

Some researchers also investigated the effect of co-inhibiting other molecules in combination with HO-1 in CML cells, which are discussed in the following paragraphs.

Ma et al. aimed to investigate the correlation between NHE1 and HO-1. They found that inhibiting NHE1 reduces HO-1 expression. They reported that NHE1 activity resulted in PKC-β phosphorylation and P38-MAPK pathway activation, which in turn upregulated HO-1. Also, combinational inhibition of NHE1 and HO-1 plus IM treatment significantly increased the apoptosis in IM-resistant CML cells. HO-1 inhibition increased caspase 3 activity and expression. Incubation of IM-resistant cells of patients with ZnPP increased sensitivity towards IM. Moreover, following a reduction in PH_i_ by NHE1 inhibition, HO-1 expression was decreased [73].

The association between HO-1 and HDACs was investigated in another study. Following HO-1 silencing, mRNA and protein expression levels of HDAC1, HDAC2, HDAC3, HDAC4, and HDAC7 decreased. Combined blockade of HO-1 and HDACs resulted in significantly higher apoptosis compared to each blockade alone. Following HO-1 upregulation, ROS generation in cells decreased and HDAC expression increased. It seems that HO-1 overexpression upregulates HDAC expression by altering ROS levels in CML cells [80].

In another study of combinational therapy, a group of investigators designed and synthesized a series of hybrid compounds with the ability to co-inhibit both HO-1 and tyrosine kinase called HO-1/TKI hybrids. The hybrids were able to decrease HO-1 expression, induce ROS formation in cells, and elevate apoptosis, thus overcoming IM resistance. The data indicate that combinational therapy of IM and HO-1 silencing might help eliminate IM-resistant CML cells [104]. The same group in their subsequent research designed a new series of hybrids by replacing the IM with a Nilotinib (NIL)-like segment. Replacing IM inhibitor with NIL inhibitor was predicted to exert higher toxicity. Therefore, the effect of treatment with these compounds was evaluated on NIL-resistant and sensitive cells. The hybrids efficiently induced apoptosis; however, did not significantly improve the apoptosis of cells compared to NIL or IM treatment, and only a moderate blocking of HO-1 was noted. They suggested that HO-1 is not involved in NIL resistance in CML [105].

To conclude, inhibition of HO-1 reduces CML IM resistance, and the above-discussed studies confirm this and simultaneous inhibition of HO-1 combined with NHE1, HDACs, as well as simultaneous inhibition of HO-1 and BCR-ABL by hybrid compounds (HO-1+IM), have promising results and could be helpful in the removal of IM-resistant cells. While, it appears that HO-1 might not be involved in NIL resistance, therefore HO-1 blockade combined with NIL treatment may not present a favorable outcome. Also, HO-1 overexpression by stromal cells induces resistance to chemotherapy by inducing BcL-2 expression in CML cells. Treatment with IM in combination with HO-1 inhibition is a helpful option for IM-resistant patients and provides new hope; that future clinical trials can help confirm this data.

### Targeting HO-1 in AML

Due to the increased expression of HO-1 in AML, as well as the high probability of resistance to chemotherapy in AML, many researchers have tried to target HO-1 in combination with Ara-c and DNR treatment.

Accordingly, Zhe et al. showed that silencing HO-1 enhanced apoptosis in response to Ara-c treatment. Moreover, following HO-1 silencing HIF-1α and GLUT1 expressions were decreased [30], which are considered proteins responsible for chemoresistance [106].

Similarly, HO-1 downregulation in AML cells increased apoptosis rates in another study. They proposed that while Ara-c and DNR induce apoptosis by enhancing ROS formation, HO-1 protects AML cells from oxidative stress and induces chemoresistance [82].

In the support of previously mentioned studies, following HO-1 silencing, in response to DNR treatment, survival of cells decreased, which was associated with the upregulation of caspase 3, 8, and 9. Furthermore, survival time was significantly higher in mice inoculated with HO-1-silenced CML cells compared to mice injected with HO-1-expressing cells [83].

Consistently, HO-1 silencing in AML cells enhanced Ara-c-induced apoptosis. Also, murine models injected with HO-1-silenced AML cells had increased survival, less tissue infiltration, and less enlarged spleen compared to HO-1-non-silenced AML cell receivers. It was discovered that HO-1 activates the JNK/c-jun pathway to inhibit apoptosis in AML cells. Therefore, combinational inhibition of the JNK/c-jun pathway and HO-1 highly increased apoptosis compared to AML cells which received only HO-1 inhibition [84].

Herman et al. also showed that the growth of AML cells was inhibited in response to PEG-ZnPP or SMA-ZnPP treatment and successful apoptosis was induced in a dose-dependent manner. It was found that ZnPP treatment synergizes with Ara-c in inducing apoptosis. Also, mice injected with SMA-ZnPP pretreated cells had higher event-free survival compared to mice injected with untreated cells [92].

The same results were achieved by another study reporting that inhibition of HO-1 decreased cell survival in response to Ara-c, whereas Bach1 inhibition, increased HO-1 expression and increased survival [107].

In another study, the effect of silencing HO-1 on apoptosis induction was evaluated. HO-1 silencing in combination with DNR treatment induced significantly higher growth inhibition in AML cells compared to DNR alone. Also, it was associated with increased cleavage of caspase 3, 8, and 9 and increased caspase 12, cytoplasmic cytochrome c, and cleaved nuclear poly (ADP-ribose) polymerase (PARP) in AML cells. Also, Ca^2+^ accumulation and ROS generation in the combinational group was higher compared to other groups. Thus, HO-1 inhibition results in both mitochondrial and endoplasmic-mediated apoptosis induction [108].

Due to the uniformity of the data, it can be confirmed that HO-1 overexpression is associated with Ara-c and DNR resistance, and blocking its activity synergizes with these chemotherapeutics in eliminating AML cells.

On the other hand, other studies evaluated the effect of targeting HO-1 in combination with other anticancer drugs.

Valenzuela et al., report that treating AML cells with Arsenic trioxide (ATO) induces ROS formation which results in HO-1 overexpression mediated by Nrf2 activation. Following the HO-1 blockade, cell sensitivity to ATO increased. They found that treating AML and Acute Promyelocytic Leukemia (APL) cells with All-Trans Retinoic Acid (ATRA) inhibits Nrf2 and HO-1 function, thus, resulting in improved cell death [109].

According to a recent study, blocking HO-1 in FLT-ITD^+^ AML cells synergizes with quizartinib in inducing apoptosis in TKI-resistant/-sensitive FLT3-ITD^+^ cells. The study reports that ROS (produced by the constitutive activity of FLT3-ITD) induces Nrf2, which then induces HO-1 expression resulting in quizartinib resistance [88].

According to the findings of Cheng et al., HO-1 contributes in Panobinostat resistance as well. Following the downregulation of HO-1, the sensitivity of AML cells to panobinostat increased, while GFI-1 silencing induced Panobinostat resistance. It was discovered that HO-1, upregulated due to the GFI-1 low expression by a PI3K/AKT-mediated pathway, is a potential Panobinostat-resistance inducer in AML [90].

Ma et al. evaluated the efficacy of HO-1 blocking in combination with ursolic acid (UA), a natural pentacyclic triterpenoid acid used as a traditional antileukemic drug in china, in AML cells. Following the blockade of HO-1, cells became more susceptible to apoptosis induction by UA. They also investigated the combinational effect of HO-1 silencing and UA treatment and found increased apoptosis compared to controls. Also, mice injected with AML cells treated with ZnPP plus UA or Ara-c showed similar results in the rate of apoptosis with an increased survival rate [110].

As discussed previously, HO-1 contributes to the immune evasion of AML cells. Zhang et al. investigated the mechanism behind the subject matter. They found that HO-1 knocked-down AML cells had lower survival compared to cells with upregulated HO-1 when cocultured with NK cells. They found that HO-1 induced Silent information regulator 1 (Sirt1) expression, an HDAC, which in turn downregulated CD48 expression, therefore inhibiting NK-cell anti-tumor functions. While silencing HO-1 decreased Sirt1 expression. Moreover, the results of the in vivo studies in mice models also confirmed the role of HO-1 in promoting tumor growth and suppressing NK-cell cytotoxic activity [94].

As discussed previously, HO-1 inhibition is highly beneficial in reducing resistance to chemotherapy. Altogether, HO-1 synergizes with several chemotherapeutics and anticancer drugs including Ara-c and DNR, ATO, HDAC inhibitors, TKIs, and UA. Moreover, HO-1 silencing, improves the anti-tumor responses of NK cells, indicating its future immunotherapeutic application.

#### The role of HO-1 in TNF-induced apoptosis resistance in AML

In addition to chemotherapy drugs, HO-1 appears to be involved in inducing resistance to TNF treatment. TNF by binding to TNFR1 and TNFR2 has both cellular proliferation and cellular death-inducing functions [111]. In physiologic conditions, TNF-TNFR signaling via NF-КB expression induces the expression of multiple anti-apoptotic genes and inhibits cell death. However, following NF-КB inhibition, cells such as macrophages and monocytes are highly sensitive to TNF treatment [112–114].

Rushworth et al. studied the association between NF-КB and HO-1 in AML cells. They reported increased expression of HO-1 in AML cells following NF-КB inhibition and found that NF-КB suppresses HO-1 expression. Therefore, following NF-КB inhibition, HO-1 is upregulated and induces a second line of apoptosis resistance. Moreover, they found that the effect of HO-1 combined inhibition with NF-КB was higher than silencing each factor alone [32]. According to this study, NF-КB inhibition should be accompanied by HO-1 inhibition to prevent HO-1 induction following NF-КB inhibition and efficiently kill AML cells.

The same group in their subsequent study evaluated the role of HO-1 in resistance to TNF-induced apoptosis. They found that treating NF-КB-inhibited AML cell lines with TNF, induces Nrf2 expression which results in HO-1 expression, therefore, preventing TNF-mediated apoptosis. They found that HO-1 or Nrf2 blocking sensitizes AML cell lines to TNF-induced apoptosis by a caspase-dependent pathway [115].

Another study was also conducted by the same researchers, reporting that treating AML cells with TNF induces NF-КB, which in turn increases the expression of FLIC-inhibiting protein-long form (FLIP_L_). FLIP_L_ expression results in TNF resistance while silencing it improves cell death. Moreover, following FLIP silencing, HO-1 is upregulated providing another obstacle for apoptosis induction. Therefore, Rushworth et al. inhibited FLIP_L_ in combination with HO-1 and found further sensitivity to TNF-induced apoptosis compared to the inhibition of either of the genes separately. They suggested that FLIP_L_ inhibition should be accompanied by HO-1 inhibition to efficiently eliminate AML cells [116].

It can be concluded that AML cells use two different mechanisms to escape TNF-induced apoptosis. First, by FLIP_L_ induction by NF-КB-mediated mechanism, and the second one is the induction of Nrf2, when NF-КB is blocked, which in turn induces HO-1 expression. Therefore, the inhibition of NF-КB or FLIP_L_ should be accompanied by HO-1 inhibition to achieve the highest levels of apoptosis in response to TNF treatment.

### Targeting HO-1 in CLL and ATL

A few studies have investigated the role of HO-1 in CLL, reviewed here.

Matrix Metalloproteinase-9 (MMP-9) is a crucial enzyme that plays a vital role in several biological processes. Through proteolytic cleavage, MMP-9 can control how the extracellular matrix (ECM) remodels by degrading numerous ECM proteins [117]. According to a study by Jimenez et al., MMP-9 is upregulated in response to ATO or Fludarabine treatment and correlates with ATO and Fludarabine resistance in CLL. Also, silencing MMP9 overcomes the resistance to the mentioned chemotherapeutics. Therefore, making it a potential target for CLL treatment [118].

According to their subsequent study, HO-1 downregulates MMP-9 expression by inhibiting the activation of the P38 MAPK-c-jun pathway. Therefore, sensitizing CLL cells to ATO treatment. Moreover, the same study reported that HO-1 upregulation decreased the viability of untreated and ATO-treated CLL cells. While *HMOX1* silencing reversed this effect. *HMOX1* silencing was correlated with the increase in Bcl-xL and a decrease in Bim and Bax expression. The data indicate that HO-1 has pro-apoptotic properties in treating CLL with ATO [119].

The same results will likely be obtained for fludarabine treatment of CLL, as MMP9 is increased in CLL cells under the influence of fludarabine treatment, and this increase could be mediated by HO-1. Thus, silencing HO-1 in combination with Fludarabine or ATO treatment in CLL cells appears to be unfavorable [118, 119].

The data is in the support of a study about the role of HO-1 in Adult T-cell Leukemia (ATL), which states that HO-1 increases the anticancer effects of bortezomib (a proteasome inhibitor) in ATL cells. Following HO-1 induction, the bortezomib-induced apoptosis was increased. On the other hand, HO-1 inhibiting led to decreased apoptosis in response to bortezomib in ATL cells [120].

However, another study reports that HO-1 silencing synergizes with auranofin, an FDA-approved rheumatoid arthritis (RA) drug, in inducing apoptosis in CLL cells, which was mediated by ROS induction [121]. A supporting research indicated that HO-1 is indirectly involved in the biogenesis of mitochondria. According to this study, CLL cells possess increased metabolism resulting in increased ROS formation, which in turn induces HO-1 expression, as an anti-oxidant enzyme. Next, HO-1 induces Mitochondrial Transcription Factor A (TFAM) which results in mitochondrial biogenesis and subsequent ROS generation, forming a cycle. The study suggests that simultaneous targeting of HO-1 and TFAM could present a new therapeutic approach [122].

Therefore, HO-1 appears to play a variety of (pro- and anti-apoptotic) roles in CLL, and the exact role of HO-1 in CLL remains to be discovered. According to the data, HO-1 targetting in CLL combined with ATO treatment and probably Fludarabine is not favorable. Despite the scarce number of studies, the same results are available regarding the effect of treating ATL cells with bortezomib. However, silencing HO-1 sensitizes CLL cells to auranofin treatment.

### Targeting HO-1 in ALL

In this section, the role of HO-1 in causing resistance to several chemotherapeutics in ALL is discussed.

In a study, following treating ALL cells with SMA-ZnPP and PEG-ZnPP, the growth of cells was inhibited compared to normal BM cells. Also, following this treatment, IM-resistant cells became more susceptible to apoptosis in response to IM treatment. Combinational inhibition of HO-1 with bendamustine (an alkylating agent) treatment induced high levels of apoptosis and growth inhibition. Also, a combination of HO-1 inhibition and IM treatment formed a synergism to efficiently inhibit the growth of ALL cells [95].

Another study reported that following HO-1 silencing, the growth of CD34^+^ ALL cells was inhibited, and an increase in caspase-3 cleavage was seen. As stated previously IK6 activates HO-1 resulting in increased proliferation, decreased apoptosis, and increased resistance to IM. Following HO-1 silencing or blocking, sensitivity to IM increased. It was discovered that IK6 through STAT5 activation, induces HO-1 in ALL cells [99].

It appears that HO-1 induction by IK6 through STAT5, also by other pathways causes ALL resistance to bendamustine and IM. Thus, bendamustine and IM treatment combined with HO-1 blocking are suggested in ALL.

As we previously mentioned, HO-1 is expressed in BMSCs of ALL patients in addition to ALL cells, therefore a study aimed to target HO-1 in BMSCs. Enhanced viability of ALL cells was also observed in response to vincristine treatment following coculture with HO-1 up-regulated BMSCs of ALL patients. In contrast, HO-1-silenced BMSCs coculturing with ALL cells had a higher apoptotic effect and upregulated expression of cleaved caspase-3 and 9. They suggested that HO-1 induces cell cycle arrest in G0/G1, thus, resulting in decreased apoptosis in response to vincristine treatment. Moreover, xenograft models of ALL, showed that HO-1 overexpression resulted in more tumor burden and BM infiltration by ALL cells compared to mice injected with low HO-1 expressing cells. Also, vincristine-resistant patients' BMSCs and upregulated BMSCs produced more VEGF by an HO-1-dependent pathway, which was associated with vincristine resistance. It was suggested that HO-1 enhanced VEGF expression and induced vincristine resistance in ALL cells through the PI3K/PKB pathway [96]. Therefore, HO-1 is also involved in vincristine resistance as well.

The expression of HDAC4/5 and Smad7 are considered poor prognostic markers and are associated with decreased apoptosis of cells. A study on ALL patients reported that high HO-1-expressing cells had increased Suppressor of Mothers against Decapentaplegic 7 (Smad7) and HDAC4/5 expression compared to low HO-1-expressing cells. Interestingly, following treatment with ZnPP or LMK-235(an HDAC 4/5 inhibitor), decreased Smad7 was seen in ALL cells. Also, LMK235-treated ALL cells exhibited increased apoptosis mediated by PKB pathway inhibition. Additionally, HO-1 upregulation led to an increase in BcL-2 and caspase 8 expression and activation of the PKB pathway. However Smad7 silencing reversed these effects. The study suggested that HO-1 and Smad7 (functioning downstream of HO-1) overexpression induce PKB phosphorylation which results in apoptosis resistance in response to HDAC4/5 inhibitors while inhibiting HO-1 or Smad7 overcomes this [123].

Concluding from the last two studies, it was discovered that HO-1 utilizes the PKB pathway in BMSCs to induce VEGF production resulting in vincristine resistance, while in ALL cells, this pathway is used to prevent apoptosis induced by HDAC inhibitors.

In conclusion, HO-1 in ALL appears to prevent apoptosis in response to various drugs such as HDAC inhibitors, vincristine, bendamustine, and IM mediated by different pathways. HO-1 inhibition in ALL, according to the studies discussed earlier, increases apoptosis and sensitivity to drugs.

## Conclusions

According to the literature, it has been shown that HO-1 exerts crucial functions in various malignancies. HO-1 is an essential enzyme in the heme catabolism pathway. Through the HO-1 function, the heme molecule is broken into biliverdin, CO, and Fe^2+^ [20]. Recent studies investigated the role, the expression pattern, as well as the targeting effect of HO-1 in MDS and various leukemias.

Several studies have shown a strong association between increased HO-1 expression and more severe disease in MDS patients as well as in vivo studies performed on MDS mouse models. Hence, HO-1 has a high potential to determine the prognosis of MDS patients. Moreover, HO-1 through various molecules including EZH2 (mediated by pRB-EBF) and DNMT1 reduces the expression of anti-oncogenes such as p15, p53, and p16, which in turn, enervates the efficacy of AZA and decitabine treatment. Moreover, HO-1 overexpression by BM macrophages is an informant of a decreased OS and can help determine the prognosis of MDS patients alongside other factors. Therefore, studies indicate that HO-1 mostly induces chemoresistance in MDS by inhibiting anti-tumor gene demethylation. Due to the presence of a hyper-methylated profile of anti-oncogene in MDS [124, 125], the common use of drugs such as AZA and decitabine, and also, due to the proven role of HO-1 in preventing the demethylation of anti-oncogenes such as p15 and p16, as well as the induction of BcL-2, DNMT1, EZH2 and cell cycle progression by HO-1, it can be said with high confidence that HO-1inhibition in MDS (at least in high-risk patients) is beneficial and increases the effectiveness of the mentioned drugs and could even reduce the required dose to alleviate side effects.

In the case of CML, the data from several studies were consistent enough to conclude that HO-1 is overexpressed in CML. In addition, this overexpression is associated with increased resistance to IM which was mediated by various pathways. Reduction of ROS formation [80], HDAC induction [80], and autophagy [10] are several pathways that have been investigated. While HO-1 inhibition overcomes IM resistance in CML cells. Certainly, HO-1 has other roles in inducing resistance that future research will help elucidate. Therefore, treating CML with IM combined with HO-1 targeting appears to be promising.

The overexpression of HO-1 in AML is a fact, which is more pronounced in Ara-c-resistant cells than in Ara-c-sensitive cells. HO-1 expression in patients' cells is associated with a poor prognosis. HO-1 is also expressed in LSCs of AML patients. Therefore, it can be a suitable target for eliminating MRD. Additionally, its role in determining the outcome of HSCT is Noteworthy. HO-1 appears to inhibit apoptosis by its role in decreasing ROS formation because its inhibition is associated with increased intracellular ROS accumulation and induction of apoptosis. In vivo studies also confirm the role of HO-1 in tumor progression. HO-1 overexpression is also involved in resistance to ATO, quizartinib, UA, TNF, and HDAC inhibitors.

Based on the very few studies in CLL, HO-1 appears to have a proapoptotic as well as an anti-apoptotic role in CLL. However, its exact role in the CLL needs to be further studied. Also, targeting HO-1 in ATL not only is not beneficial but also worsens the response to bortezomib treatment.

HO-1 in ALL has increased expression and is coexpressed with IK6, Smad7, and HDAC4/5, which are considered markers of poor prognosis. Also, a special polymorphism of HO-1 ((GT)n repeat in *HMOX1* promoter) is found to be associated with a poor prognosis [100]. Elevated HO-1 expression, observed in BMSCs of ALL patients, induces VEGF production through the PI3K/AKT pathway, thereby, reducing apoptosis and increasing the resistance of ALL cells to vincristine, while the same pathway is utilized in ALL cells to prevent apoptosis induced by HDAC inhibitors. [96]. Thus, HO-1 is expressed by LSCs, BMSCs, and leukemic cells of ALL patients and is an ideal target along with vincristine, bendamustine, IM, and HDAC inhibitors.

In addition to the results achieved, we also discovered some new points: First, by summarizing various articles and examining the different mechanisms used by HO-1, we found that HO-1 in each malignancy utilizes specific mechanisms to induce resistance to various chemotherapeutics. For instance, the main mechanism used in MDS to induce resistance to decitabine and AZA is the inhibition of demethylation of tumor suppressor genes by HO-1. However, these mechanisms in AML, ALL, and CLL have a wider range, which includes the prevention of ROS production, induction of autophagy, VEGF production, etc. With this in mind, treatments can be selected in a targeted way to achieve the best therapeutic response.

Next, is the expression of HO-1 by LSCs. According to studies, ALL [95], CML [103], and AML [84] LSCs express HO-1. This can change treatment approaches in a way that can improve patients' recovery by eliminating MRD.

In the meantime, a series of sections remained unknown. Future studies should examine these gaps.

First, is the effects of *HMOX1* gene polymorphisms on the course of various HMs. In a study regarding the role of HO-1 gene polymorphisms in ALL, it was discovered that the presence of certain polymorphisms could affect the prognosis of patients. Therefore, determining the effect of common mutations of *HMOX1* in MDS and leukemias could be of prognostic value. Second, is determining the role of HO-1 in CLL. HO-1 appears to have both pro-apoptotic as well as anti-apoptotic roles in CLL. Further studies with larger statistical populations are needed to determine the exact role of HO-1 in CLL. Third, most of the studies in the field of ALL were in the Ph^+^ ALL group. Therefore, the exact role of HO-1 in Ph^−^ ALL is yet to be fully understood. It is predicted that HO-1 induces resistance to treatment by different mechanisms (independent of BCR::ABL) in these cells.

Overall, HO-1 has increased expression in MDS, AML, CML, and ALL, which is associated with resistance to several chemotherapeutics. Also, HO-1 has prognostic value in these disorders. This resistance is mediated by a variety of pathways, including inhibition of gene demethylation in MDS, inhibition of ROS production in AML, as well as various pathways in CML and ALL, such as autophagy induction, and immune evasion. The exact role of HO-1 in CLL is still unclear. In addition to HO-1 expression by malignant cells, BMSCs of ALL, and AML patients express HO-1 which has prognostic and therapeutic value. Moreover, combined inhibition of HO-1 with several chemotherapeutics improves the elimination of leukemic cells. Clinical trials are needed to determine the effectiveness of HO-1 inhibition in reversing chemoresistance in different leukemia.

## Data Availability

Not applicable.

## Abbreviations

aGVHD: Acute graft versus host disease
ALL: Acute lymphoblastic leukemia
AML: Acute myeloid leukemia
Ara-c: Cytarabine
AREs: Antioxidant response elements
ATL: Adult T-cell leukemia
ATO: Arsenic trioxide
AZA: 5-Azacytidine
Bak: Bcl-2 homologous antagonist/killer
Bax: Bcl-2 associated X
Bcl-2: B-cell lymphoma 2
Bcl-Xl: B-cell lymphoma-extra-large
BM: Bone marrow
BMMCs: Bone marrow mononuclear cells
BMSCs: Bone marrow stromal cells
CLL: Chronic lymphocytic leukemia
CML: Chronic myeloid leukemia
DNMT1: DNA methyltransferase-1
DNR: Daunorubicin
EZH2: Enhancer of zest homolog 2
FLIP: FLIC-inhibiting protein
*FLT3-* ITD: Fms-like tyrosine kinase receptor 3 gene-internal tandem duplication
GFI-1: Growth factor independent-1
GLUT1: GlucoseTransporter-1
HDAC: Histone deacetylase
HIF-1α: Hypoxia-inducible factor-1 alpha
HMs: Hematological malignancies
HO-1: Heme oxygenase-1
HSCT: Hematopoietic stem cell transplantation
Hsp32: Heat shock protein 32
IM: Imatinib
JNK: C-Jun N-terminal kinases
Keap1: Kelch-like ECH-associated protein 1
LSC: Leukemic stem cell
MAPK: Mitogen-activated protein kinase
MDS: Myelodysplastic syndromes
MRD: Minimal residual disease
mTOR: Mammalian target of rapamycin
NADPH: Nicotinamide adenine dinucleotide phosphate
NF-КB: Nuclear factor kappa-light-chain-enhancer of activated B cells
NHE1: Na^+^–H^+^ exchanger 1
NIL: Nilotinib
NILI: Nilotinib inhibitor
NK cells: Natural killer cells
Nrf2: NF-E2-related factor 2
OS: Overall survival
PB: Peripheral blood
Ph: Philadelphia chromosome
PKB: Protein kinase B
PKC: Protein kinase C
pRB-EBF: Retinoblastoma protein-early B-cell factors
ROS: Reactive oxygen species
Smad7: Suppressor of mothers against decapentaplegic 7
STAT5: Signal transducer and activator of transcription 5
StRE: Stress-responsive element
TFAM: Mitochondrial transcription factor A
TKI: Tyrosine kinase inhibitor
TNF: Tumor necrosis factor
UA: Ursolic acid
VEGF: Vascular endothelial growth factor
ZnBG: Zinc deuterophyrin IX 2, 4-Bis-ethylene glycol
ZNPP: Zinc protoporphyrin
